# Application of “living high-training low” enhances cardiac function and skeletal muscle oxygenation during submaximal exercises in athletes

**DOI:** 10.20463/jenb.2017.0064

**Published:** 2017-03-31

**Authors:** Hun-Young Park, Sang-Seok Nam

**Affiliations:** 1 Physical Activity and Performance Institute, Konkuk University, Seoul Republic of Korea; 2 Department of Sports Medicine, Kyung Hee University, Yongin Republic of Korea; 3 Republic of Korea

**Keywords:** Living high-training low, Cardiac function, Skeletal muscle oxygenation, Submaximal exercise, Athletes

## Abstract

**[Purpose]:**

The aim of this study was to determine the efficiency of the application of living high-training low (LHTL) on cardiac function and skeletal muscle oxygenation during submaximal exercises compared with that of living low-training low (LLTL) in athletes.

**[Methods]:**

Male middle- and long-distance runners (n = 20) were randomly assigned into the LLTL group (n = 10, living at 1000-m altitude and training at 700-1330-m altitude) and the LHTL group (n = 10, living at simulated 3000-m altitude and training at 700-1330-m altitude). Their cardiac function and skeletal muscle oxygenation during submaximal exercises at sea level before and after training at each environmental condition were evaluated.

**[Results]:**

There was a significant interaction only in the stroke volume (SV); however, the heart rate (HR), end-diastolic volume (EDV), and end-systolic volume (ESV) showed significant main effects within time; HR and SV significantly increased during training in the LHTL group compared with those in the LLTL group. EDV also significantly increased during training in both groups; however, the LHTL group had a higher increase than the LLTL group. ESV significantly increased during training in the LLTL group. There was no significant difference in the ejection fraction and cardiac output. The skeletal muscle oxygen profiles had no significant differences but improved in the LHTL group compared with those in the LLTL group.

**[Conclusion]:**

LHTL can yield favorable effects on cardiac function by improving the HR, SV, EDV, and ESV during submaximal exercises compared with LLTL in athletes.

## INTRODUCTION

Altitude and hypoxic training are common among many personal or team sports athletes and recommended by many coaches and scientists for normoxic exercise performance^1^. 

Generally, normoxic exercise performance can be improved using three methods through altitude and hypoxic training^2^. First, living high-training high (LHTH) was the first design of living and training at 1500-4000 m in altitude environments that enhances erythropoiesis and exercise performance at sea level. However, LHTH has a major limitation, that is, failure to perform training of the same intensities (e.g., running speed), as compared with the sea-level training^3^. Several studies demonstrated that absolute training intensities during continuous and interval workout significantly decreased at 2500 m, as compared with the sea-level training^4, 5^. 

Second, intermittent hypoxic training (IHT) commonly involves shorter hypoxic exposure (approximately two to five sessions per week of < 3 h) and lesser cost, effort, and time than other altitude and hypoxic training methods^6^. However, the efficacy of IHT for the enhancement of normoxic exercise performance is more of a matter of debate owing to the methodological differences, including in the dose of hypoxic stimulus, type and intensity of training, participant training status, and time-point of the measurement of exercise performance^7-9^. 

Third, living high-training low (LHTL) was developed for potential improvements in the limitation of LHTH. LHTL simultaneously offers athletes the beneficial effects of acclimation in altitudes and hypoxic residing (e.g., erythropoiesis) and training at or near sea level (i.e., maintenance of training intensity). Therefore, despite ongoing debate regarding the efficiency of altitude and hypoxic training methods for normoxic exercise performance or the nature of its underlying mechanism, LHTL in altitude and hypoxic training is widely recognized as the “gold-standard” for normoxic exercise performance via erythropoiesis and maximal oxygen consumption (VO_2max_) in athletes^1, 4, 10^. 

Normoxic exercise performance is highly related to a number of factors that can be altered through various altitude and hypoxic training methods, including increases in the exercise economy^11^, acid-base response in the muscle^29^, skeletal muscle oxygenation^27^, cardiac function^13^, vascular function^30^, and microcirculation^31^ as well as oxygen transport capacity (e.g., erythropoiesis)^1^ and VO_2max_^3^. Considering previous studies related to the improvement of athletic performance via LHTL, Levine and Stray-Gunderson^32^ assumed that acclimatization to a moderate altitude (2,500 m) plus training at a low altitude (1,250 m) (LHTL) improves sea-level performance in well-trained runners more than an equivalent sea-level or altitude control. As a result, 4 wk of applying LHTL improves sea-level running performance in trained runners owing to an increase in the red cell mass volume and VO_2max_ and maintenance of sea-level training velocities, most likely accounting for the increase in the velocity of the VO_2max_. Further, Robach et al^21^ tested whether LHTL enhances aerobic performance in athletes and whether any positive effect may last for up to 2 wk after LHTL. They concluded that LHTL may stimulate red cell production, without any concurrent amelioration of aerobic performance, and the absence of any prolonged benefit after LHTL suggests that this LHTL model cannot be recommended for long-term purposes. Siebenmann et al^33^ verified LHTL efficacy in athletic performance with the use of a placebo-controlled, double-blinded design; they tested the hypothesis that LHTL-related improvements in endurance performance are mediated through physiological mechanisms and not through a placebo effect. Their results indicate that LHTL, using normobaric hypoxia, may not improve endurance performance at sea level or moderate altitude more than conventional training. 

Similarly, most previous studies related to LHTL for the improvement of exercise performance at sea level have focused on the oxygen transport capacity of the blood (e.g., erythropoiesis), VO_2max_, and energy metabolism during submaximal exercises; however, the results of these studies are not consistent. Thus, it is very valuable to investigate the changes in the cardiac function and skeletal muscle oxygenation, which are very important factors for normoxic exercise performance, via LHTL application. 

Therefore, using a randomized and controlled design, this study aimed to investigate the efficiency of the application of LHTL on cardiac function and skeletal muscle oxygenation during submaximal exercises at sea level in athletes. 

## METHODS

### Participants

The participants were male middle- and long-distance runners (n = 20) who are registered in the Korea Association Athletic Federation. The participants were randomly assigned by a blinded investigator to either the living low-training low (LLTL; n = 10) or LHTL (n = 10) group according to their initial physical fitness level. They did not participate in any exercise and training program at natural or simulated altitude conditions in the previous 6 months; they were non-smokers and did not have any history of musculoskeletal, cardiovascular, or pulmonary diseases. They received information about the purpose and process of this study, and all participants provided informed consent prior to the start of the study. All procedures followed were in accordance with the ethical standards of the responsible committee on human experimentation and with the Helsinki Declaration principles. 

### Experimental design

Twenty male athletes in the LLTL group (n = 10) and LHTL group (n = 10) performed various training sessions daily, except in Sundays for 4 wk. Training sessions were conducted for more than 4 h and consisted of dawn (warm-up, 90-100 bpm and 60 min jogging, 130-160 bpm), morning (warm-up, 90-100 bpm; 5 times 150-m accelerated running, 160-180 bpm; and 6 times 1000-m running, 170-190 bpm), and afternoon exercises (warmup, 90-100 bpm; 5 times 300-m accelerated running, 165-190 bpm; and hill running exercise, 160-180 bpm). Maximal heart rate (HRmax) was determined using the predicted HRmax formula (Male = 206 – 0.69 × age)^12^. 

We designed this study to verify the effectiveness of LHTL on cardiac function and skeletal muscle oxygenation during submaximal exercises compared with those of LLTL. Accordingly, we analyzed the cardiac function parameters and skeletal muscle oxygenation profiles during the submaximal exercises on a bicycle with an exercise intensity corresponding to 130 watts and 60 rpm for 30 min. The exercise intensity with 130 watts and 60 rpm corresponds to the lactate threshold (LT) intensity^13^. 

The living condition in the LLTL group was at a 1000-m altitude at a resort located in Taebaek City, Republic of Korea. A 3000-m altitude (14.5% O_2_) for the living condition in the LHTL group was simulated using normobaric hypoxic environments by introducing nitrogen into the resort, using a nitrogen generator (Separation & Filter Energy Technology Cooperation, Korea); this generator has the capacity to simulate normobaric hypoxic environments for altitudes of up to 6000 m (9.7% O_2_). The LLTL group resided at a 1000-m altitude location under comfort conditions similar to those in the LHTL group. Moreover, training in all groups was performed at 700-1330-m altitudes. The temperature within the resort was maintained at 24 ± 2oC and the humidity at 60 ± 5% for all the conditions. 

### Measurements

#### Body composition

All participants fasted overnight prior to the measurement of their body composition (i.e., height, weight, and body fat percentage). They wore lightweight clothing and were asked to remove any metal items. An X-SCAN PLUS (Jawon medical, Korea) was used to measure height and body composition^29, 31^. 

#### Cardiac function during the submaximal exercises

Cardiac function parameters (heart rateHR, stroke volumeSV, end-diastolic volumeEDV, end-systolic volumeESV, ejection fractionEF, and cardiac outputCO) were measured before and after training while the participants performed the submaximal exercises on a bicycle (Monark Exercise AB, Vansbro, Sweden) for 30 min at sea level (i.e., normoxic conditions); the summation values were used as the measurement values, except for EF, in which the average value was used. The HR, SV, EDV, ESV, EF, and CO were assessed noninvasively using a thoracic bioelectrical impedance device (PhysioFlow PF-05 Lab1, Manatec Biomedical, Paris, France), which has been previously shown to provide reliable results in healthy men and patients with chronic pulmonary disease^14, 15^. The electrodes were positioned on the forehead, neck, xiphoid process, and lower ribs on the left side, avoiding the abdominal muscles, as these positions were suggested to be appropriate for human participants^11^. 

#### Skeletal muscle oxygenation during the submaximal exercises

The skeletal muscle oxygenation profiles (concentration of oxy-hemoglobin and myoglobinO_2_Hb, concentration of deoxy-hemoglobin and myoglobin lHHb, and tissue oxygen indexTOI) were measured before and after training while the participants performed the submaximal exercises on a bicycle (Monark Exercise AB, Vansbro, Sweden) for 30 min at sea level (i.e., normoxic conditions); the average values were used as the measurement values. The skeletal muscle oxygenation profiles of the left vastus lateralis were evaluated using a commercially available near-infrared spectroscopy (NIRS) system (Hamamatsu NIRO 200, Hamamatsu Photonics, Japan). The intensity of the incident and transmitted light was recorded continuously with the relevant specific extinction coefficients used for the online estimation of the changes (Δ) from the baseline concentrations of the O_2_Hb, HHb, and totalhemoglobin + myoglobin (Hb_TOT_)^16^. From these values, a measurement value of the skeletal muscle oxygenation was used to calculate the O_2_Hb, HHb, and TOI (calculated by 100 × O_2_Hb/Hb_TOT_). 

### Statistical analysis

Statistical analyses were conducted using the SPSS 23.0 (IBM Corp., Armonk, USA) for windows. All data were presented as means ± SDs. A two-way repeated analysis of variance (ANOVA) was used to determine the interaction and main effects within time and between the groups. A post-hoc test within time and between the groups was used with a paired and independent t-test. A priori, the level of significance was set at 0.05. 

## RESULTS

The participants’ compliance and adherence to the study design were 100% in all groups, based on the daily activity records. The characteristics of the selected athletes between the two groups showed no significant difference (Table 1). 

**Table 1:** Participant characteristics

Variable	LLTL group	LHTL group
N	10	10
Natural or simulated altitude	Living 1000 m	Living 3000 m
	Training 700-1330 m	Training 700-1330 m
Age (yrs)	17.0 ± 1.3	17.6 ± 1.5
Height (cm)	173.1 ± 4.8	174.8 ± 7.4
Weight (kg)	55.8 ± 5.5	60.3 ± 6.9
Body fat (%)	11.9 ± 2.1	12.5 ± 1.5

LLTL, living low-training low; LHTL, living high-training low.

### Cardiac function during the submaximal exercises

**Figure 1.**
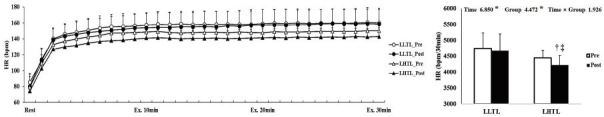
Changes in heart rate (HR) over 30 min (a.) and summation (b.) during submaximal exercise on a bicycle at Pre and Post in LLTL and LHTL group. The bars indicate the mean ± S.D. *: significant interaction or main effect, †: significant difference between Pre and Post in each group, ‡: significant difference between LLTL and LHTL group in each time.

**Figure 2.**
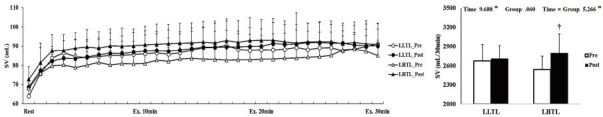
Changes in stroke volume (SV) over 30 min (a.) and summation (b.) during submaximal exercise on a bicycle at Pre and Post in LLTL and LHTL group. The bars indicate the mean ± S.D. *: significant interaction or main effect, †: significant difference between Pre and Post in each group.

**Figure 3.**
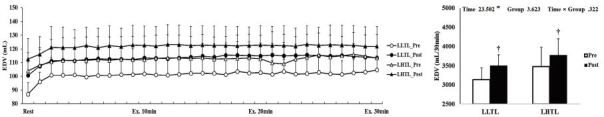
Changes in end-diastolic volume (EDV) over 30 min (a.) and summation (b.) during submaximal exercise on a bicycle at Pre and Post in LLTL and LHTL group. The bars indicate the mean ± S.D. *: significant interaction or main effect, †: significant difference between Pre and Post in each group.

**Figure 4.**
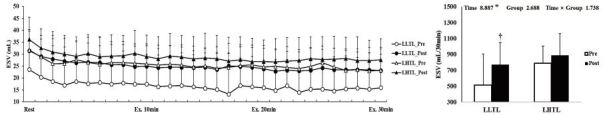
Changes in end-systolic volume (ESV) over 30 min (a.) and summation (b.) during submaximal exercise on a bicycle at Pre and Post in LLTL and LHTL group. The bars indicate the mean ± S.D. *: significant interaction or main effect, †: significant difference between Pre and Post in each group.

**Figure 5.**
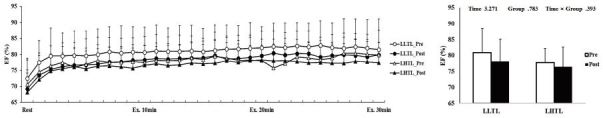
Changes in ejection fraction (EF) over 30 min (a.) and average (b.) during submaximal exercise on a bicycle at Pre and Post in LLTL and LHTL group. The bars indicate the mean ± S.D.

**Figure 6.**
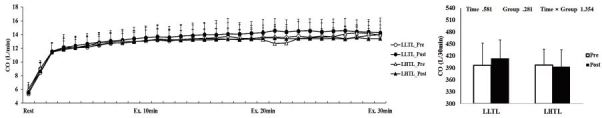
Changes in cardiac output (CO) over 30 min (a.) and summation (b.) during submaximal exercise on a bicycle at Pre and Post in LLTL and LHTL group. The bars indicate the mean ± S.D.

Following the 4 wk of training at each environmental condition, there were significant interactions (time × group) in the SV; further, the increase in the SV was significantly higher during training in the LHTL group than in the LLTL group (Figure 2). HR showed a significant main effect within time and between the groups; HR significantly increased during training in the LHTL group and had a higher value after training than that in the LLTL group (Figure 1). EDV and ESV showed a significant main effect within time; EDV significantly increased in both groups, while ESV significantly increased only in the LLTL group (Figures 3 and 4). There was no significant difference in the EF and CO (Figures 5 and 6). 

### Skeletal muscle oxygenation during the exercises

**Figure 7.**
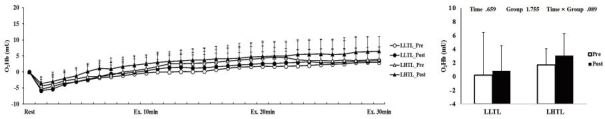
Changes in concentration of oxy-hemoglobin and myoglobin (O2Hb) over 30 min (a.) and average (b.) during submaximal exercise on a bicycle at Pre and Post in LLTL and LHTL group. The bars indicate the mean ± S.D.

**Figure 8.**
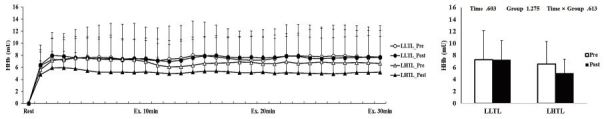
Changes in concentration of deoxy-hemoglobin and myoglobin (HHb) over 30 min (a.) and average (b.) during submaximal exercise on a bicycle at Pre and Post in LLTL and LHTL group. The bars indicate the mean ± S.D.

**Figure 9.**
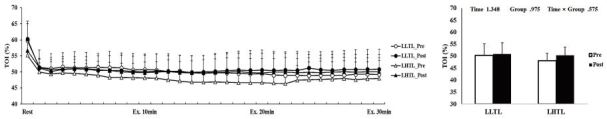
Changes in concentration of tissue oxygenation index (TOI) over 30 min (a.) and average (b.) during submaximal exercise on a bicycle at Pre and Post in LLTL and LHTL group. The bars indicate the mean ± S.D.

Unlike what we expected, the skeletal muscle oxygenation profiles did not show any significant difference; however, it improved in the LHTL group; O_2_Hb and TOI increased and HHb decreased in the LHTL group compared with those in the LLTL group (Figures 7-9). 

## DISCUSSION

A number of studies have been conducted on the efficacy of the application of altitude and hypoxic training interventions on normoxic exercise performance in athletes. These studies reported positive and negative results owing to the physiological characteristics (e.g., body weight, percent of body fat, sex differences, and individual differences) and training conditions (e.g., method, intensity, frequency, duration, and time of training)^13^. 

Altitude and hypoxic training have positive effects on exercise economy, acid-base response in the blood and muscle, erythropoiesis, glycolytic enzyme, muscle buffer capacity, VO_2max_, and erythropoiesis^1, 2, 6, 13, 17, 18^. Together with its positive effects, altitude and hypoxic training also negatively affect blood viscosity, muscle blood flow, cardiac output, HRmax, protein synthesis, and Na^+^-K^+^- ATPase activity and decrease training quality and quantity^19-22^. Similarly, normoxic exercise performance is highly related to a number of factors that can be altered through altitude and hypoxic training methods, including increases in the exercise economy^11^, acid-base response in the muscle^29^, skeletal muscle oxygenation^27^, cardiac function^13^, vascular function^30^, and microcirculation^31^ as well as oxygen transport capacity (e.g., erythropoiesis)1 and VO_2max_^3^. 

Most studies related to altitude and hypoxic training, specifically LHTL, for the improvement of exercise performance at sea level have focused on the oxygen transport capacity of the blood (e.g., erythropoiesis), VO_2max_, and energy metabolism during submaximal exercises; the results of these studies are not consistent^21, 32, 33^. Therefore, we investigated the efficiency of the application of LHTL on cardiac function and skeletal muscle oxygenation during submaximal exercises at sea level in athletes. As a result, the 4-wk LHTL was sufficient to enhance the cardiac function and can potentially improve the skeletal muscle oxygenation in athletes. 

LHTL enhances erythropoiesis and oxygen transport and utilization capacities by residing at high-altitude or hypoxic environments and performing high-intensity trainings at or near sea level^1, 5, 6, 13, 19, 21^. Cardiac function is the most important variable for oxygen transport capacity, which is a determinant of the improvement of normoxic exercise performance^11, 23^. In general, LHTL increases energy availability that stimulates the parasympathetic nervous system through activation of β-adrenergic receptors in the cardiac muscles and improves SV with enhanced left ventricular contractility^24^. Further, several studies have found that LHTL led to higher left ventricular contractility and oxygen utilization in the cardiac and skeletal muscles^24-26^. To verify the effect of LHTL on cardiac function, which is one of the major determinants of performance, Liu et al^24^ divided 21 well-trained triathletes into control (n = 10, living and training at sea level) and LHTL groups (living at 1980-m altitude ≥ 12 h/d and training at sea level); the cardiac function of these triathletes was examined via Doppler echocardiography before and at the end of the 2-wk program. As a result, the left ventricular end-systolic diameter of the LHTL group was lower than that of the control group. Further, the shortening fraction and EF in the LHTL group increased by 9% and 17%, respectively; their SV and CO also increased. They suggested that LHTL improved the systolic function underlined by the incremented left ventricular contractility, which might be associated with increased adrenergic receptors or improved myocardial energy utilization. 

Our study revealed that LHTL significantly decreased the HR and increased the SV during the submaximal exercises. EDV significantly increased in both groups; however, ESV significantly increased only in the LLTL group. Although LLTL and LHTL increased the preload (the pressure that stretches the right or left ventricle of the heart to its greatest dimensions under variable physiologic demands), only LHTL increased the afterload (the pressure in the wall of the left ventricle during ejection). In other words, ventricular relaxation depending upon the EDV is caused by enhanced venous return and left ventricular volume through exercise training, and not by stimulation of hypoxic conditions. However, LHTL should increase ventricular contractility and SV. Our results are consistent with those of several studies showing that the improvement in the ventricular contractility through ESV and SV should result from the activation of β-adrenergic receptors and increased energy efficiency in the cardiac muscles^24-26^. Furthermore, the decrease in the HR in the LHTL group during the submaximal exercises would be linked to the decreased sympathetic nerve activity and increased parasympathetic nerve activity, resulting in the activation of α2-adrenergic receptors^11, 24^. Conversely, our data showing no change in the CO after LHTL attributes to the changes in the HR and SV (the actual effects on the CO), in contrast with those of previous studies^13, 24^. 

Nevertheless, we verified the adaptation effect of LHTL on oxygen utilization capacity in the mitochondria during the submaximal exercises by measuring the skeletal muscle oxygenation profiles of the left vastus lateralis using the commercially available NIRS system^27, 28^. Although the O_2_Hb, HHb, and TOI did not significantly change, all skeletal muscle oxygenation profiles increased during the submaximal exercises via LHTL. Consequently, we verified that LHTL enhanced the oxygen utilization capacity in the mitochondria in the skeletal muscle. 

## CONCLUSIONS

The present study demonstrates that LHTL had a positive effect on cardiac function and can potentially improve the skeletal muscle oxygenation during submaximal exercises in athletes compared with LLTL. In addition, our study provides some novel possibilities regarding greater exercise performance via enhanced cardiac function and skeletal muscles in athletes. Therefore, more facilities that can apply LHTL are needed to improve the competitiveness of Korean elite athletes. 
